# Three-Dimensional Reconstruction and Navigation Systems in Endoscopic Ultrasound Procedures: A Comprehensive Review

**DOI:** 10.3390/diagnostics16020366

**Published:** 2026-01-22

**Authors:** Eyad Gadour, Bogdan Miutescu, Bodour Raheem, Abed Al-Lehibi, Abdulrahman Alfadda, Ana Maria Ghiuchici, Antonio Facciorusso

**Affiliations:** 1 Multiorgan Transplant Centre of Excellence, Liver Transplantation Unit, King Fahad Specialist Hospital, Dammam 32252, Saudi Arabia; 2 Department of Medicine, Zamzam University College, Khartoum 11113, Sudan; 3 Department of Gastroenterology and Hepatology, “Victor Babes” University of Medicine and Pharmacy, 300041 Timișoara, Romania; 4 Adanced Regional Research Center of Gastroenterology and Hepatology, “Victor Babes” University of Medicine and Pharmacy, 300041 Timișoara, Romania; 5 Department of Gastroenterology, King Salman Medical City, Al Madinah Al Munawwarah 42319, Saudi Arabia; 6 Department of Medicine, Faculty of Medicine, Al-Imam Mohammad Ibn Saud Islamic University, King Fahad Medical City, Riyadh 1223, Saudi Arabia; aallehibi@kfmc.med.sa; 7 Advanced Therapeutic Endoscopy Unit, King Faisal Specialist Hospital and Research Centre, Riyadh 1223, Saudi Arabia; 8 Department of Surgical and Medical Sciences, Section of Gastroenterology, University of Foggia, 71122 Foggia, Italy

**Keywords:** three-dimensional reconstruction, navigation system, endoscopic ultrasound, gastrointestinal, prostate fluid collection, augmented reality

## Abstract

Three-dimensional (3D) reconstruction of ultrasound (US) images represents a novel advancement that has been extensively explored over the past three decades. This technique enables endoscopists to perform more detailed and enhanced visualizations of anatomical structures, which is not feasible using traditional ultrasound methods. The reconstructed images also facilitate navigation during endoscopy-guided procedures, such as fine-needle aspiration. Furthermore, augmented reality (AR) algorithms can overlay the reconstructed images with real-time anatomical images, thereby enhancing clinician performance during these procedures. Current evidence suggests that 3D ultrasound reconstruction has already been widely implemented in various clinical imaging studies. However, its application for generating procedural guidance and augmented reality overlays remains in the early research stages and has not yet achieved widespread adoption. Existing pre-clinical evidence suggests that 3D reconstruction has significant potential to enhance clinician performance in various ultrasound-guided procedures.

## 1. Introduction

One notable advancement in endoscopic ultrasound (EUS) technology is the development of three-dimensional (3D) systems [1]. These systems enable the reconstruction of 3D images of the gastrointestinal tract (GIT) and adjacent organs [2], allowing endoscopists to perform detailed analyses of the overall anatomical structure, thereby facilitating informed diagnoses and decisions and the creation of comprehensive management plans [3]. Generating high-quality 3D images necessitates acquiring multiple two-dimensional (2D) ultrasound images to reduce noise and motion artifacts [2]. This process is often challenging and time-intensive [4]. This narrative review primarily focuses on the technological and methodological aspects of using three-dimensional reconstruction and navigation systems in endoscopic ultrasound procedures. Emphasis is placed on early-stage applications and technical developments rather than on clinical outcomes or efficacy. To address these challenges, 3D-EUS systems equipped with convex-type endoscopes have been developed. These systems employ an electromagnetic tracking system to ascertain the probe’s position and subsequently compile 3D images of the anatomical site. Furthermore, they enable the concurrent integration of 3D images into the constructed volume, thereby yielding high-quality 3D images [2]. This narrative review aims to synthesize and summarize the current evidence regarding the state of the art on 3D reconstruction and navigation systems in EUS procedures.

## 2. Methodology

A non-systematic electronic search was conducted on two databases, namely PubMed and Google Scholar. The keywords utilized for each search were based on the various subthemes of the manuscript. The following keywords were used in the search: (“Endoscopic ultrasound”, “3-dimensional reconstruction” OR “3-dimensional ultrasound” OR “Image registration” OR “Image Registration”). The review included all references that provided vital information on the topic.

## 3. EUS Imaging/Procedures Fundamentals

### 3.1. Ultrasound in Endoscopy

The introduction of ultrasound in endoscopy marked a significant advancement in 21st-century medical care, fundamentally transforming the practice of endoscopy [4]. This innovative system, known as EUS, serves as a robust diagnostic instrument, enabling sonographers to visualize the distinct sonographic layers of various luminal structures in accordance with the respective histologic tissue planes [4]. Subsequent technological advancements have facilitated the integration of a catheter equipped with intractable needles, thereby enabling fine-needle aspiration of identified target lesions [5]. The amalgamation of EUS with fine-needle aspiration (FNA) has allowed for the definitive histologic staging of GIT lesions [5]. Recent advancements have included the development of 3D reconstruction using high-quality images generated via EUS systems [6].

Fundamental EUS systems comprise endosonoscopes, categorized into two primary types: radial and linear scanning endosonoscopes [7]. Radial scanning endosonoscopes (RSEs) were the initial equipment developed for performing EUS [8]. In this system, the ultrasound transducer features a proximal endoscopic portal that captures an oblique view of the lumen while navigating the gastrointestinal tract [9]. As the ultrasound transducer rotates, it provides a 360° cross-sectional image of the lumen perpendicular to the axis or shaft of the endoscope [10]. The viewing frequencies of this system alternate between 7.5 and 12 MHz. Additionally, the transducer is encased in a water-filling balloon system, which mitigates the challenges of imaging through an air-filled lumen [9].

The other type of system employed in endoscopic imaging is linear array endosonoscopes. These systems furnish longitudinal images along the axis of the endosonoscope within a 100° to 180° arc [11,12]. The balloon system and the optic for luminal viewing are analogous to those of the radial endosonoscope. However, the transducers of linear array scopes have two fundamental designs: mechanical and electrical linear array systems [12]. Mechanical array systems incorporate a rotating transducer that reflects waves in a linear array. Conversely, electronic systems employ a transducer oriented along the endoscope’s axis, transmitting sound waves laterally [7]. The frequencies of these systems typically range between 5 and 7.5 MHz. Given that images from linear array scopes are aligned along the axis of the scope, they are the only probes integrated with EUS-FNA systems [12]. This integration facilitates the simultaneous visualization of the target lesions and the endoscopic needle, thereby reducing the incidence of adverse events to less than 5% [13].

High-frequency ultrasound probe sonography (HFUPS) represents an advanced system that employs a miniature rotating ultrasound transducer integrated at the catheter tip, which can be inserted through the working channel of a standard endoscope [14]. This system produces high-quality, detailed images at frequencies ranging from 10 to 20 MHz [15,16]. A primary advantage of these probes is their ability to effectively visualize intramural or small mucosal lesions, as the miniprobes can be positioned directly over the target lesion through endoscopic guidance. Furthermore, the high-quality, detailed images facilitate the assessment of the depth of the invasion of such lesions [16].

### 3.2. Characteristics of Imaging

EUS has been extensively adopted as a critical diagnostic modality; however, image artifacts continue to pose significant challenges, particularly in diagnosing and managing diseases affecting hollow organs [17]. These artifacts, which encompass motion blur, pixel saturation, specular reflections, and motion and defocus blur, as well as fluid, bubble, and debris interference, can substantially corrupt EUS video frames [17]. Such artifacts impede the visualization of tissues during reconstruction and adversely affect the post-analysis of the generated images and videos [17,18]. It is imperative for clinicians to accurately identify all EUS artifacts to prevent the misinterpretation of images, which could lead to erroneous diagnoses [19]. A specific imaging artifact inherent to EUS is speckle noise, which complicates the identification of tissue layer boundaries within the AR wall due to slight differences in acoustic impedance [20]. Although specific post-processing techniques for EUS images aim to eliminate these speckles, they frequently fail to reveal structures obscured by speckle noise. Nevertheless, methods such as elevational angular compounding can reduce speckle interference by integrating multiple frames, thereby alleviating the limitations associated with single-frame analysis [20].

### 3.3. Navigation and Reconstruction Implications

Most artifacts in EUS arise from the fundamental operating principles of the technology. These artifacts are generally undesirable as they compromise the quality of the generated images, thereby complicating the accurate interpretation and diagnosis of the underlying condition [18]. The influence of these artifacts extends beyond mere image interpretation, affecting navigation and the reconstruction of three-dimensional images [18,21]. It is, therefore, advisable for clinicians to be cognizant of these artifacts and consider their implications when making diagnoses or planning interventions.

## 4. 3D Reconstruction in EUS

### Computational Procedures in EUS

The final 3D image in EUS is reconstructed from a sequence of 2D images, determined based on the position of each frame [2]. The position of the video frame is derived from the endoscope’s angle of rotation, utilizing various algorithms and matrices [2]. Given that this process can be executed rapidly, i.e., up to 15 to 20 times per second, it enables the real-time display of 3D images during scanning [2]. It is crucial to acknowledge that 3D reconstruction is a complex process, involving several stages of development. These stages encompass camera calibration, depth estimation, and point cloud reconstruction and registration. Notably, camera calibration and depth estimation are most pertinent to the accuracy of reconstruction and are, thus, examined in detail in this study.

Camera calibration is essential for aligning the pixel coordinates of a camera’s image with the actual spatial location of the corresponding point in the captured scene. Precise camera calibration is a fundamental requirement for accurately synthesizing spatial information from acquired images [22]. Various methodologies exist for performing camera calibration. The 3D calibration of a camera using a 2D image target was initially developed by Tsai et al. [23], and subsequent advancements have been made by researchers seeking to optimize the calibration process [24]. Ramirez et al. employed a triangulation method to compute 3D coordinates from corresponding 2D points [25]. This method was refined to address errors arising from digitalization and lens distortion, which occur due to the exclusion of several camera parameters [25].

Depth estimation constitutes a critical aspect of 3D reconstruction. In the context of EUS, using a single endoscope probe necessitates the application of monocular depth estimation (MDE). MDE has been implemented through various technologies, including Simultaneous Localization and Mapping (SLAM), Structure from Motion (SFM), and Computer Vision (CV) methodologies. The SLAM system is an algorithm that concurrently determines the position of a robot and constructs a representation of the explored environment by processing information from the image flow [26,27]. It is widely used due to its reliability, robustness, and comprehensive performance [26]. In 3D reconstruction, SLAM is indispensable as it allows the computer system to accurately locate the EUS transducer, thereby facilitating the creation of anatomically precise reconstructions based on real-time image data from each location [28]. The reconstruction of images from monocular input views is a fundamental challenge inherent to Monocular Computer Vision. In scenarios where monocular input views are limited, reconstruction methods must rely on priors over object shapes. Learning-based methods (LBMs) have been developed to address this limitation [29], as they employ algorithms to encode the necessary priors from available data [29]. SFM is a computational technique used to reconstruct images from unordered image sets. Various SFM strategies have been proposed for image reconstruction, including hierarchical, incremental, and global approaches [30]. Among these, incremental SFM is the most prevalent strategy [30]. SFM is particularly useful in situations where images are provided without a specific order [22]. To enhance 3D image reconstruction, some researchers have explored integrating these technologies with machine learning, yielding promising preliminary results regarding its feasibility [31,32,33].

## 5. Navigation Systems in EUS/Endoscopy

### 5.1. General Navigation Frameworks in Optical Endoscopy

To ensure the safety and efficacy of endoscopic procedures, it is imperative to employ a method for navigating flexible endoscopes through the lumens of various organs [34]. For many endoscopists, the use of endoscopes is non-intuitive and presents numerous challenges, which are further exacerbated in endoscopy-guided procedures, including surgeries. Consequently, practical navigation algorithms are essential to address these challenges [34]. Seamless navigation during endoscopy necessitates three types of information: the environmental knowledge of the cavity or lumen being examined, the direction of the scope’s movement, and the target location where the scope should conclude [22]. In most endoscopic procedures, visual navigation is employed. Through this approach, endoscopists interpret the images transmitted via the endoscope to ascertain the anatomical position of the endoscope and subsequently guide it to the desired anatomical location [34]. There are two proposed principal subdivisions of this technique: lumen centralization and visual odometry [34]. In lumen centralization, endoscopists identify a target within the image, typically the center of the lumen, which is the deepest and darkest area in the image [35]. Steering the endoscope involves maintaining the target at the center of the image. In visual odometry, the motion between images is determined [36]. In flexible endoscopy, this motion generally corresponds to the camera’s displacement, which is analogous to the endoscope’s displacement since the camera is positioned at the tip of the endoscope [34]. Endoscopists then utilize virtual landmarks, which provide both environmental information and define the target using data about their relative distance, location, or intensity properties [37].

### 5.2. Registration Techniques and Augmented Reality Overlays

Image registration entails determining the geometric transformation necessary to align corresponding points in one view of an image with those in another view of the same or a different image [38]. This process is critical in endoscopy-guided interventions, serving as the primary method for integrating pre-interventional imaging with images generated during the intervention [39]. Additionally, it is essential for the diagnosis and prognosis of various conditions, as it facilitates the comparison of different images obtained from the same patient. Various techniques are employed in ultrasound image registration, including monomodal ultrasound registration [40]. This technique involves comparing one ultrasound image to another, primarily to distinguish normal anatomy from pathological states [40]. Monomodal ultrasound registration is challenged by noise and various distortions/artifacts present in ultrasound images, complicating the registration process [40]. Multi-modal registration represents another image registration technique. This approach is widely used in interventional radiology, as it facilitates the integration of images from different imaging modalities, such as computed tomography (CT), magnetic resonance imaging (MRI), and ultrasound, into a single procedure [41]. The primary challenge in multi-modal image registration is defining features that can be consistently identified across different modalities, allowing for unequivocal association [41]. Various techniques have been proposed to enhance multi-modal image registration, including manual-based, feature-based, and intensity-based registration methods [41]. With the advent of machine learning, deep learning methods utilizing convolutional neural networks (CNNs) have been proposed to guide the image registration process, as they can learn from the images. CNNs can learn and extract data from highly distorted images and match them across different modalities [41].

Through image registration and 3D reconstruction, various imaging modalities can be seamlessly integrated into numerous procedures in real time. Augmented reality (AR) overlays facilitate the fusion of 3D-reconstructed images with 2D live camera images in real time. The use of AR overlays is not widespread in 3D-EUS procedures but is in endoscopic surgeries.

### 5.3. Existing Applications in EUS

The principal challenge in navigation during EUS procedures arises from the limited field of view and the flexibility of endoscopes, which complicate the accurate determination of instrument locations by clinicians [42]. To mitigate this issue, various navigation systems have been developed to enhance real-time navigation during EUS. Estépar and colleagues introduced a navigation system specifically designed for transgastric access procedures [42]. This system, known as IRGUS (Image-Registered Gastroscopic Ultrasound), employs pre-operative MRI and CT data to provide contextual information regarding the location of the endoscopic tip. Using an electromagnetic tracker, the system identifies the location of the EUS tip and the ultrasound plane, subsequently establishing the real-time position of the EUS tip based on pre-operative imaging data [42]. In a separate study, Gruionu et al. developed a Fusion Imaging (FI) system for navigation during AR endoscopic procedures [43]. This FI system incorporates an electromagnetic tracking system for spatial positioning, a navigation catheter within the endoscope’s working channel, and an active marker on the patient’s chest [43]. The system integrates three-dimensional reconstructed models of pre-operative CT images to guide the endoscope probe toward the target lesion, followed by a standard EUS-guided fine-needle aspiration (FNA) procedure. Following model simulation, the authors demonstrated the system’s feasibility for use in real patients, suggesting its potential to enhance navigation and the targeting of lesions in complex anatomical locations [43]. Vosburgh et al. demonstrated the feasibility of a similar system; however, in this system, the navigation catheter was externally affixed to the working channel of the endoscope [44]. They demonstrated that the IRGUS system was twice as effective as EUS in identifying and localizing individual structures within the GIT [44].

An ongoing clinical trial (NCT06798545) is examining an EUS navigation system [45]. This innovative navigation system provides EUS guidance through a mechanism analogous to the Global Positioning System (GPS), applied to a three-dimensional reconstructed model of the AR. The researchers propose that this navigation system will reduce the duration of the procedure and facilitate comprehensive imaging of the pancreas and associated lesions [45].

## 6. Incorporation of Navigation Aids and 3D Imaging

### 6.1. Integration of 3D-EUS with CT/MRI for Anatomical Context

Through applying multi-modal registration, ultrasound (US) images can be effectively integrated with those obtained from MRI and CT. Despite its potential, multi-modal image registration has not yet been extensively applied for various gastrointestinal tumors. Initially, the strategy involved employing spatially tracked 2D ultrasound images to facilitate image alignment in instances where tumors and lesions were not visible via ultrasound [46]. The process of registration with CT/MRI images is simplified through 3D reconstruction, as 3D images provide more structural detail compared to a single 2D ultrasound image [46]. Xing et al. introduced a 3D ultrasound-CT/MRI registration framework specifically for percutaneous focal liver tumor ablation procedures, achieving the successful registration of 3D ultrasound to MRI image pairs in 11 out of 12 patients [46]. Subsequently, HE proposed a similar yet automated framework for CT-3D ultrasound registration [47].

### 6.2. Real-Time Overlays for Needle Guidance and Lesion Targeting

Accurate needle placement under ultrasound guidance is a fundamental aspect of numerous diagnostic and therapeutic procedures in contemporary medicine. Using ultrasound facilitates the visualization of the needle tip during insertion, thereby enabling precise needle placement, even in diminutive target lesions [48]. AR has been effectively employed to guide needles during vascular access, with the initial findings indicating that AR increased the completion time of procedures in pre-clinical studies [49]. Regarding needle biopsy, AR has been under investigation for over two decades. Early trials explored the use of AR displays to guide needle biopsies of phantoms [50,51]. These studies aim to provide clinicians with greater degrees of freedom while simultaneously enhancing patient comfort, as AR-guided needle biopsies necessitate fewer injections to reach target lesions [51]. Although research on AR-guided needle interventions is diverse, the majority of studies remain pre-clinical in nature [51,52,53,54,55,56], with a limited number of studies being conducted in clinical settings [56].

### 6.3. Effect on Targeting Accuracy, Effectiveness, and Safety

Preliminary investigations suggest that AR overlay enhances the efficacy of various endoscopy-guided needle procedures. Initial experiments conducted on phantoms demonstrated that AR guidance significantly reduced deviation from the target site compared to conventional display methods (1.62 mm versus 2.48 mm, *p* < 0.02) [50]. Subsequent studies have corroborated these findings; however, the mean deviation still exhibits considerable variability across different studies, indicating a lack of consistency among various models [51,52]. The most notable performance metric shown to improve with AR-integrated needle tracking is the success rate. Preliminary studies, often conducted on phantom models or in controlled experimental conditions, have reported a 100% success rate for AR-guided needle procedures. It is important to note that these findings reflect early-stage research and may not yet be generalizable to broader clinical practice [55,56].

## 7. Three-Dimensional Pre-Clinical Validation and Benchmarking

### 7.1. Robotic Needle Placement

While many studies in this section focus on percutaneous or general ultrasound-guided interventions, their findings offer valuable knowledge applicable to EUS by highlighting shared principles and challenges, though differences in modality-specific techniques and limitations should be acknowledged.

Boctor et al. evaluated the efficacy of 3D-guided robotic needle placement [57]. Their study employed both ex vivo bovine liver models and live animal models. The findings indicated that, within the ex vivo liver model, the 3D ultrasound (US) image-guided robotic system demonstrated superior accuracy in needle placement compared to manual insertion [48]. However, in the live animal model, respiratory movements significantly compromised needle accuracy, leading to the failure of the model to reach the target lesion. Accurate needle placement was only achieved by controlling respiration through anesthesia [49]. In a separate study, Abayazid et al. assessed ultrasound-guided 3D needle steering using a robotic system in curved phantoms. Their results showed that the system could effectively target lesions with a minimum radius of 3 mm, achieving a mean targeting error ranging from 1.29 to 1.82 mm [58]. In biological tissues, the system attained a mean targeting error of 0.38 ± 0.19 mm [59].

### 7.2. Needle Tracking Algorithms

Zhao et al. conducted a simulation to assess various biopsy needle localization and tracking methods [60]. The study examined four distinct algorithms: parallel integral projection (PIP), random Hough transform (RHT), principal component analysis (PCA) and ROI-RK (ROI-based RANSAC and Kalman filter) [60]. Among these algorithms, ROI-RK demonstrated superior performance, achieving precise localization and tracking of the biopsy needle in both 3D and 4D contexts with an accuracy of 1.4 mm [60]. Furthermore, this level of accuracy was attained in real time, indicating its potential for clinical application.

### 7.3. Use of Artificial Intelligence

Arif et al. employed CNNs to integrate deep learning for detecting the needle tip and for the automatic tracking of the probe location, subsequently providing feedback to the endoscopist within a 3D ultrasound image [61]. The system was evaluated using both phantom models and in vivo data. The findings indicated that the CNN system could accurately determine the needle’s orientation and position, with mean errors of 2° and 1 mm, respectively [62].

## 8. Applications in Clinical Settings and Case Studies

### 8.1. Diagnostic Interventions

The 3D-EUS has proven to be a useful complementary tool in diagnosing various gastrointestinal lesions, especially colorectal lesions [62,63,64]. In gastric cancer, initial diagnostic accuracy studies found that 3D-EUS had a high diagnostic accuracy (85.7%) in staging, superior to conventional EUS and US [62]. Futawatari et al. later used 3D-EUS to measure tumor volume in early gastric cancer and found that it was an independent risk factor for nodal metastasis [65].

In colorectal lesions, 3D-EUS has been applied for assessing colorectal tumors. Hunerbein et al. found that 3D-EUS had an accuracy of 88% in predicting tumor invasion in colorectal cancer, which was higher than that of conventional EUS (84%) but lower than that of anorectal MRI (91%) [66]. Similarly, Giovanni et al. found that using 3D reconstruction of endorectal US images increased the accuracy of T and N classification from 71.4% (observed using conventional EUS) to 88.6% [67]. The high accuracy of 3D-EUS in improving the clinical staging of colorectal cancer has been consistently shown across many settings, demonstrating its potential as a complementary imaging tool for diagnosing colorectal cancer [68]. Besides clinical staging in colorectal cancer, 3D-EUS has been applied in diagnosing fecal incontinence. However, Wasserberg et al. found that, even with the introduction of 3D-EUS, there was no improvement between the imaging results and the clinical findings, as both healthy and sick patients had similar clinical scores after 3D-EUS examination [69].

### 8.2. Skill and Training Acquisition with 3D/Navigation Support

The learning curves associated with EUS procedures are typically extensive, even for seasoned endoscopists [70]. In contrast, studies on 3D-US-guided procedures indicate a shorter learning curve, with both novice and experienced endoscopists demonstrating comparable performance and accuracy during the initial stages [43]. However, in other ultrasound-guided procedures, such as anesthesia injection, a learning curve persists, with experts completing the procedures more swiftly than novices [71].

### 8.3. Current Limitations

Several limitations have been identified in the application of 3D reconstruction and AR overlays for guiding multiple endoscopic procedures. One notable limitation is that 3D reconstructed images and AR overlays may convey misleading depth perception, potentially hindering visualization during procedures [72]. Another observed limitation is the prolonged workflow associated with dynamic 3D reconstruction, particularly when employing AR overlays. This issue arises because most EUS systems, although capable of displaying 2D images in real time, do not facilitate the volumetric acquisition of the generated images. Consequently, since volumetric acquisition is not feasible, the images must first be stored before being exported for processing and reconstruction, resulting in approximately 25 min of extended downtime. Furthermore, this constraint implies that endoscopists cannot freely maneuver the ultrasound probe during examination, as such movement would disrupt the reconstructed 3D environment [73].

## 9. Future Directions

### 9.1. Deformation-Aware Real-Time Navigation

Deformation in ultrasound images arises when there is a discrepancy in pressure between the ultrasound transducer and the organ or tissue examined. Such deformations distort individual two-dimensional images, thereby impeding the precise reconstruction of the three-dimensional image of the organ [74]. Various methodologies have been proposed to address this issue. Burcher et al. introduced a finite element method (FEM) model to predict deformation in both lateral and axial directions [75]. Jiang et al. proposed a deformation-aware robotic system that adjusts and corrects the contact force based on vessel stiffness [74]. Despite these initial proposals, it remains necessary to further evaluate these developed robotic systems in real clinical settings to ascertain their efficacy. Additionally, further research is required to create other deformation-aware systems capable of providing real-time feedback to facilitate corrections during free-hand endoscopy.

### 9.2. AI-Driven Speckle-Adapted Reconstruction

Li et al. introduced a deep learning approach for mitigating speckle noise in three-dimensional B-mode ultrasound imaging [76]. Their model achieved a 50% reduction in errors attributed to speckles during 3D reconstruction [76]. Similarly, Chen et al. developed an ultrasound reconstruction technique leveraging deep learning, which inherently corrects for speckles during reconstruction [77]. As is common for much of the research on deep learning, these studies remain in the preliminary stages. Further investigation is required to determine how these algorithms can be effectively integrated into real-time diagnostic processes for various pathologies within clinical environments.

### 9.3. Dual-Modality Navigation (Optical and EUS)

Dual-mode photoacoustic/ultrasound endoscopy (ePAUS) has been proposed as a method for achieving dual-modality navigation during endoscopic procedures [78,79]. Pre-clinical studies have demonstrated the feasibility of this approach in animal models, suggesting its potential applicability in clinical settings. However, clinical investigations have not yet been conducted. Given the promising pre-clinical evidence, it is anticipated that future studies will be approved to evaluate these modalities in clinical environments.

### 9.4. Clinical Trials Linking Navigation Aids to Outcomes

Numerous clinical trials have been registered in the United States Clinical Trials Registry, focusing on investigating navigation systems. Specifically, NCT06798545, as previously mentioned, aims to develop a navigation system for EUS analogous to the Global Positioning System (GPS) [45]. Additionally, NCT06441292 seeks to evaluate the accuracy of a navigation method for prostate biopsy, which is based on the fusion of ultrasound and magnetic resonance imaging (MRI) techniques [80,81,82].

## 10. Conclusions

Three-dimensional reconstruction utilizing endoscopic ultrasound images is an expanding area of research. Three-dimensional reconstruction methodologies have been extensively examined, and their accuracy has been substantiated in various studies. Current empirical evidence suggests that 3D reconstruction of ultrasound images offers enhanced anatomical detail, thereby improving the visualization of various body organs. This advancement has the potential to augment the diagnostic accuracy of numerous procedures, a domain that remains underexplored. Through 3D reconstruction, it is feasible to generate AR overlays that can be integrated into procedures in real time, providing guidance and aligning pre-operative imaging with the clinician’s current 2D images. These AR overlays help clinicians to reconcile pre-operative imaging with actual patient anatomy, which may exhibit slight variations due to patient-specific or procedural factors. Consequently, this capability may enhance diagnostic and management accuracy for various conditions, thereby warranting further investigation.

Despite promising advancements in 3D reconstruction and navigation systems in EUS, several key limitations remain that constrain their immediate clinical application. Many developments and studies remain confined to the pre-clinical stage, meaning that the findings cannot be generalized to daily patient care. Furthermore, the lack of large-scale, outcome-driven clinical trials restricts robust assessment of the benefit to patient outcomes, procedural efficiency, and diagnostic accuracy of these technologies. Ongoing technical issues such as integration complexities, real-time processing limitations, and variations in image quality prevent widespread adoption. Beyond this, cost and accessibility challenges are barriers to routine clinical implementation. These problems highlight the urgent need for continuous technological improvement, comprehensive validation, and the standardization of assessment mechanisms to provide robust clinical benefits from novel 3D-EUS systems.

While reviews have previously published on the topic, most are not up to date; thus, they do not capture recent technical developments in 3D-EUS. This comprehensive review, therefore, adds value to the current literature on 3D-EUS by providing an integrated technical synthesis of 3D reconstruction in EUS, incorporating recent developments such as the use of machine learning to address challenges and limitations of 3D-EUS, including speckle noise. By consolidating all existing and emerging advances and their clinical applications, this comprehensive review provides a contemporary reference describing both the methodological developments and clinical applications of 3D-EUS.

## Data Availability

No new data were created or analyzed in this study. Data sharing is not applicable to this article.
